# Converting lateral scanning into axial focusing to speed up three-dimensional microscopy

**DOI:** 10.1038/s41377-020-00401-9

**Published:** 2020-09-18

**Authors:** Tonmoy Chakraborty, Bingying Chen, Stephan Daetwyler, Bo-Jui Chang, Oliver Vanderpoorten, Etai Sapoznik, Clemens F. Kaminski, Tuomas P. J. Knowles, Kevin M. Dean, Reto Fiolka

**Affiliations:** 1 Department of Cell Biology, UT Southwestern Medical Center, Dallas, TX USA; 2 Department of Physics and Astronomy, University of New Mexico, Albuquerque, NM USA; 3 Department of Chemical Engineering and Biotechnology, University of Cambridge, Cambridge, CB3 0AS UK; 4 Department of Chemistry, University of Cambridge, Lensfield Road, Cambridge, CB2 1EW UK; 5 Lyda Hill Department of Bioinformatics, UT Southwestern Medical Center, Dallas, TX USA

**Keywords:** Microscopy, Light-sheet microscopy

## Abstract

In optical microscopy, the slow axial scanning rate of the objective or the sample has traditionally limited the speed of volumetric imaging. Recently, by conjugating either a movable mirror to the image plane in a remote-focusing geometry or an electrically tuneable lens (ETL) to the back focal plane, rapid axial scanning has been achieved. However, mechanical actuation of a mirror limits the axial scanning rate (usually only 10–100 Hz for piezoelectric or voice coil-based actuators), while ETLs introduce spherical and higher-order aberrations that prevent high-resolution imaging. In an effort to overcome these limitations, we introduce a novel optical design that transforms a lateral-scan motion into a spherical aberration-free axial scan that can be used for high-resolution imaging. Using a galvanometric mirror, we scan a laser beam laterally in a remote-focusing arm, which is then back-reflected from different heights of a mirror in the image space. We characterize the optical performance of this remote-focusing technique and use it to accelerate axially swept light-sheet microscopy by an order of magnitude, allowing the quantification of rapid vesicular dynamics in three dimensions. We also demonstrate resonant remote focusing at 12 kHz with a two-photon raster-scanning microscope, which allows rapid imaging of brain tissues and zebrafish cardiac dynamics with diffraction-limited resolution.

## 3D microscopy: clever mirrors reflect bioimages in a heartbeat

A new optical design based on the concept of remote focusing can benefit researchers looking to capture high-speed images of biological processes in action. While most optical microscopes refocus by mechanically adjusting the distance between the sample and the objective, remote focusing setups use movable mirrors to quickly scan the focal spot of a laser along the optical axis. Dr. Reto Fiolka at the University of Texas Southwestern Medical Center in Dallas, United States, and colleagues now demonstrate that a tweak to this concept—fixed mirrors that are scanned by a moving laser—can provide aberration-free images at millisecond time scales. Experiments revealed that combinations of step-shaped and sloped mirrors enabled samples such as beating zebrafish hearts to be imaged three-dimensionally with frame rates up to an order of magnitude higher than previous remote-focusing approaches.

## Introduction

The ability to rapidly change the focal plane of an optical imaging system is of great interest in microscopy, computer vision, and laser machining. Traditionally, re-focusing has been achieved by either mechanically moving the microscope objective or the sample under study. However, because these objects have a high mass, axial scanning becomes prohibitively rate limiting, which is especially problematic in fields such as neuroscience, which requires high-speed volumetric imaging of dynamic biological processes, including membrane voltage activity (with dynamics on the timescale of 1 ms or less)^1^ and cerebral blood flow.

A potential way to alleviate this problem is through remote focusing, which is an emerging technique where the position of the optical focus is adjusted without moving the primary objective or the sample^2,3^. This technique is primarily realized by altering the wavefront in the optical train. For example, wavefront modifications can be introduced in Fourier space (e.g. conjugate to the back focal plane of the objective) with a tuneable acoustic gradient index of refraction (TAG) lens^4,5^, an electrically tuneable lens (ETL)^6^, or a deformable mirror (DM)^7^. Impressively, TAG lenses can achieve axial scan rates of hundreds of kHz in resonantly operated devices^6,8,9^. However, to date, all ETL and TAG designs approximate only a quadratic phase function for defocusing and therefore do not account for the higher-order aberrations necessary to maintain a diffraction-limited focus. While DMs are capable of producing the more complex wavefronts required for aberration-free remote focusing, like all actuators, there are trade-offs between speed and actuator stroke: rapid DMs typically have a limited stroke in the range of one wavelength^7^, which in turn limits the achievable axial focusing range. On the other hand, DMs with large stroke actuators that are thus capable of large focus changes are typically much slower.

An alternative approach is to introduce wavefront alterations in a region of the optical train that is a conjugate to the specimen. For example, by carefully matching the pupils of an imaging objective and a remote-focusing objective, aberration-free remote focusing can be achieved by moving a small mirror at the focus of the remote objective^2,3^. If designed properly, any aberrations introduced by imaging beyond the nominal focal plane of the imaging objective are exactly compensated for by the remote-focusing objective, and diffraction-limited performance is maintained. Indeed, depending on the actuator technology, scan speeds from 10–1000 Hz have been reported. However, once again, the trade-off between the actuator stroke and bandwidth remains, and no known technology is capable of going beyond 1 kHz.

More recently, axial re-focusing on the nanosecond timescale has been reported by multiplexing laser pulses^10–13^ or through the introduction of a reverberation loop^14^. However, technological (pulse repetition rate) and photophysical (fluorescence lifetime) limitations restrict reverberation microscopy to a maximum of approximately ten focal planes. In addition, like TAG lenses and ETLs, reverberation microscopy introduces only a quadratic phase function for re-focusing, which limits it to low-resolution imaging. Finally, reverberation microscopy creates a series of differently focused laser spots with an exponentially decaying intensity. As such, it is best suited for focal planes that are spaced by one scattering mean-free path, which is typically 100 µm in brain tissue. For smaller degrees of re-focusing, very uneven illumination of the different focal planes results. Owing to these challenges, some laboratories have discontinued axial scanning altogether and instead use projection imaging^15^ or spatially encode the *Z*-position^16^. Nonetheless, the former sacrifices information in the third dimension, and the latter requires sparse samples. As such, there remains a need for a high-resolution axial scanning technology capable of reaching multi-kHz rates while avoiding spherical aberrations.

Since very fast lateral-scan technologies have been established and are readily available, including electro-optical deflectors and mirror galvanometers^17–19^, we asked ourselves if an optical system could be designed that transforms a lateral-scan motion into axial re-focusing. The simplest method we envisioned is to take the concept of aberration-free remote focusing, and instead of moving the corresponding remote mirror axially, we scan a laser spot laterally with a high-speed galvanometer over a stationary mirror. If the distance between the stationary mirror and objective lens can be made to vary in the scan direction, defocus will be introduced as is necessary for remote re-focusing. Furthermore, if the lateral-scan component is perfectly compensated for on the return path, pure axial scan motion will be obtained. Such a method would be able to harness the aforementioned high-speed scanning technologies while maintaining aberration-free remote focusing.

In this manuscript, we present two realizations of this concept: one realization capable of performing discrete axial steps and the other realization capable of continuous axial scanning. In principle, the former allows arbitrarily large axial step sizes over a finite number of steps, and the latter allows an arbitrary number and size of axial steps, albeit over a more limited scan range. We present numerical simulations and experimental measurements of both scanning technologies, and using resonant galvanometric scanning, demonstrate axial focusing at a rate of 12 kHz. We further leverage our new scanning mechanism to speed up the frame rate of axially swept light-sheet microscopy (ASLM)^20,21^ and use this form of high-resolution light-sheet microscopy to image rapid intracellular dynamics. Finally, we used this technology to perform resonant remote focusing in two-photon microscopy to image brain tissues as well as cardiac dynamics in a zebrafish embryo.

## Results

**Fig. 1:**
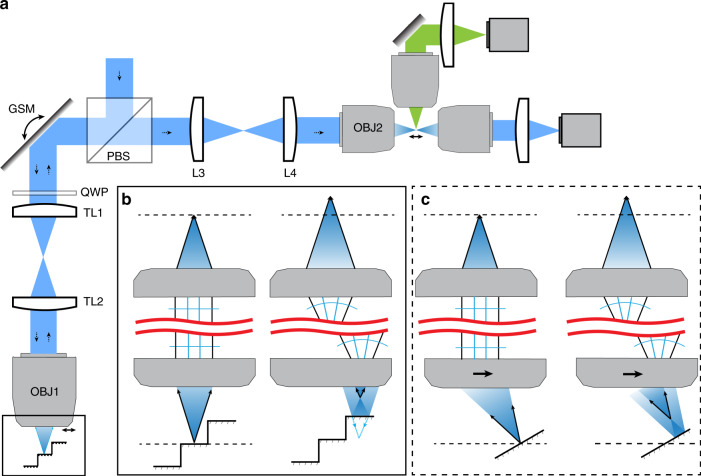
Schematic illustration of the remote-focusing approach. Setup: **a** A collimated laser beam is delivered into the setup by a beam splitter (BS) and onto a galvanometric scanning mirror (GSM), which is imaged into the back focal plane of an air objective (OBJ1). Scanning the GSM translates the focus in one dimension, as shown by the double-headed arrow in the boxed front focal space of OBJ1. A step mirror reflects the light with different amounts of defocus back into the objective, which then travels through the 4F system onto the GSM, which removes the lateral-scan motion, leaving only defocus in the wavefront. The GSM is again imaged onto the back focal plane of a water dipping objective (OBJ2). As OBJ1 and OBJ2 are pupil matched, OBJ2 forms an aberration-free image of the focus (as formed by OBJ1) in the sample space. **b** Zoomed-in view of the boxed region from **a**. The panel on the left shows the focus of the light at its nominal focus. Black arrows show returning marginal rays after reflection. Each step on the mirror results in a focus spot in the sample plane with a displaced axial position. **c** Alternative configuration with a tilted mirror that allows continuous axial scanning. Here, the remote objective OBJ1 is slightly shifted off the optical axis to create a tilted focus that is perpendicular to the mirror surface. Scanning this focus laterally results in a change in focus, as illustrated by the black arrows

Figure 1a shows a schematic representation of our experimental setup, which consists of two perpendicular arms, with a 4F telescope and an objective in each arm. The remote-focusing arm contains a galvanometric scanning mirror (GSM) and an air objective lens (OBJ1), while the other arm, which will be referred to as the illumination arm, consists of a pupil-matched water immersion objective (OBJ2). These two arms are aligned such that the GSM is conjugate to the back focal plane of both objectives. Laser foci with different axial positions emerging from OBJ2 are then observed with two imaging systems: one system for direct transmission measurements of the foci and the other to observe fluorescence in an orthogonal direction. Both imaging systems consist of water immersion objectives, tube lenses, a camera, and appropriate optical filters.

A collimated laser beam is reflected by the polarizing beam splitter into the remote-focusing arm where the beam is scanned by the GSM, which in turn produces a lateral-scan motion of the laser focus generated by OBJ1. For discrete axial re-focusing, a step mirror is placed in the focal plane of OBJ1 (Fig. 1b, left side). When the laser spot is incident on a step that is exactly in the focal plane, the back-reflected laser becomes a collimated beam in infinity space and forms a laser spot in the nominal focal plane of OBJ2. Importantly, as the returning laser beam is de-scanned by the GSM, no lateral scanning motion is imparted on the focus formed by OBJ2 (i.e. the scan motion is purely axial). If the laser beam is scanned to a step that does not coincide with the nominal focal plane, a laser focus away from the focal plane is formed (Fig. 1b, right side). The returning laser beam is now converging or diverging in infinity space, which in turn causes the laser focus formed by OBJ2 to be axially refocused as well. For an optical setup where the pupils of the two objectives have been accurately matched (in short, the image space from the air objective OBJ1 has to be de-magnified by a factor of 1.33 into the image space of the water objective OBJ2)^2,3^, aberration-free remote focusing is achieved.

For continuous axial re-focusing, the step mirror is replaced by a slightly tilted, planar mirror (Fig. 1c). The incoming laser focus is also tilted such that it is incident in a direction normal to the mirror surface. In our setup, this tilt is achieved by slightly translating OBJ1 with respect to the incoming laser beam (Supplementary Note 1 and Supplementary Figs. S1–4 and Supplementary Video 1). For a beam position where the laser focus is formed on the mirror surface, a collimated beam is formed in infinity space, and a laser spot at the nominal focal plane of OBJ2 is formed (Fig. 1c, left side). If the beam is scanned laterally, a focus away from the mirror plane is formed (Fig. 1c, right side), which in turn causes the beam in infinity space to converge or diverge and leads to an axially displaced focus formed by OBJ2. For small tilt angles, we found numerically (Supplementary Note 1) and experimentally that the focusing remains free of spherical aberrations. For the continuous Z-scanning method, the angular aperture of OBJ1 has to be larger than that of OBJ2 to permit tilting of the laser focus without sacrificing the numerical aperture of OBJ2. The achievable axial scan range scales approximately with the field of view of OBJ1 times the tangent of the mirror tilt angle. Thus, the technique benefits from objectives and scanning systems that possess a large field of view (Supplementary Note 2 and Supplementary Fig. S5). In contrast, using the step mirror, arbitrarily large axial displacements can be realized, with the caveat that wider steps are necessary for large amounts of defocus. This requirement puts practical limits on how many steps can be used for a given field of view of the remote objective (Supplementary Note 3 and Supplementary Fig. S6).

**Fig. 2:**
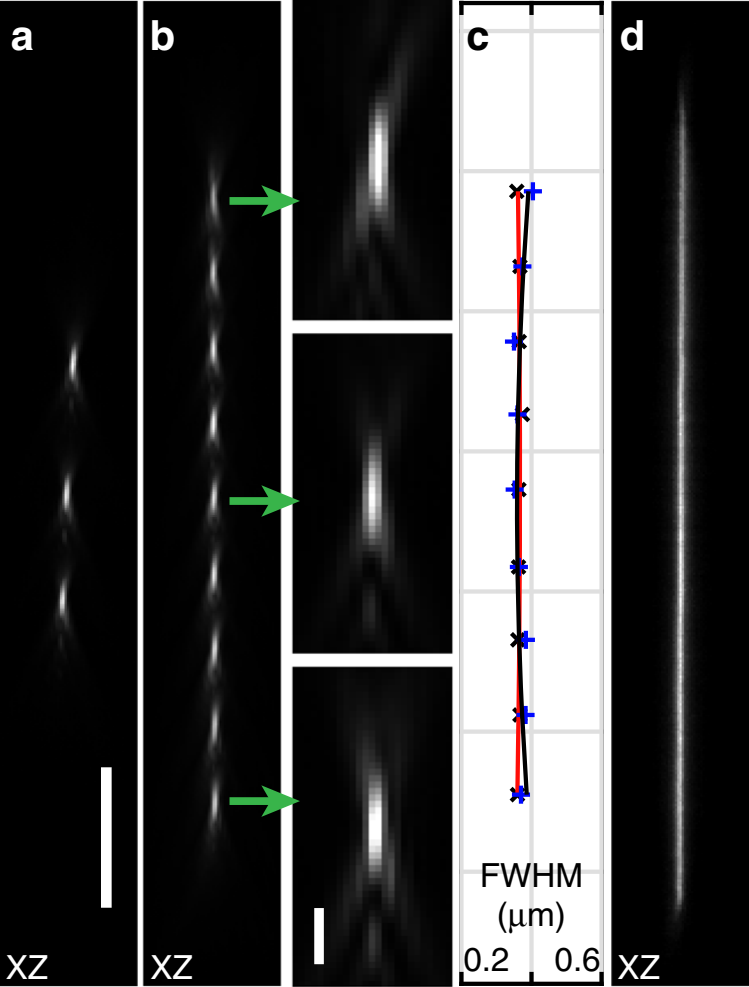
Remote focusing using a remote step mirror and a planar mirror inclined by 7.5°. **a** PSFs obtained using a remote mirror with three steps. **b** PSFs generated from discrete lateral scanning steps over a planar, tilted mirror. Green arrows depict the zoomed-in view. **c** Plot showing the full width at half maximum (FWHM) of the PSFs at different axial focus positions. The FWHM of the PSF calculated in the *X*-direction (red) and *Y*-direction (black). **d** Continuous axial scanning obtained by driving the GSM with a triangular waveform at 100 Hz using a tilted, planar mirror. Scale bar: **a**–**d** 10 µm. The scale bar in the zoomed-in image of **b** is 1 µm

To experimentally test this concept, we first measured the point spread functions (PSFs) for remote focusing using a mirror with three steps with a step height of ~6 µm. The mirror was aligned such that the middle step was located in the nominal focal plane of OBJ1 and centred in the middle of the scan range of GSM1. By consecutively positioning the laser spot on each step, we can translate the focus formed by OBJ2 to three discrete axial positions, which are shown in Fig. 2a. The optical quality of the foci corresponds closely to conventional remote focusing, i.e. translating the remote mirror mechanically in the axial direction (Supplementary Fig. S7).

To test the continuous axial focusing technique, we replaced the step mirror with a planar mirror that was tilted by 7.5° with respect to the image plane, and OBJ1 was carefully laterally translated such that the returning beam retraced the same optical path as in the non-tilted case. Scanning the GSM in discrete steps allowed us to produce a series of axially displaced laser spots that span a range of 43 µm in the *Z*-direction, as shown in Fig. 2b. Measuring the full width at half maximum of the profiles (364 ± 4.4 nm in the *X*-direction, 371 ± 16 nm in the *Y*-direction) confirmed a performance close to the diffraction limit of 305 nm for a wavelength of 488 nm and a numerical aperture of 0.8. Finally, we applied a sawtooth waveform to the GSM, which generated a linear axial line scan (Fig. 2d). In the example shown, the beam was scanned to the limit of the field of view of our scan optics; hence, field curvature effects became noticeable. As a consequence, there was a slight tilting of the foci at the end of the scan range (Supplementary Note 1 and Supplementary Fig. S8). The scan range for the discrete axial foci (shown in Fig. 2b) is over a range where the field curvature of our lateral scanning system is small, and a linear focusing response to an increment in GSM angle is obtained (Supplementary Fig. S9).

**Fig. 3:**
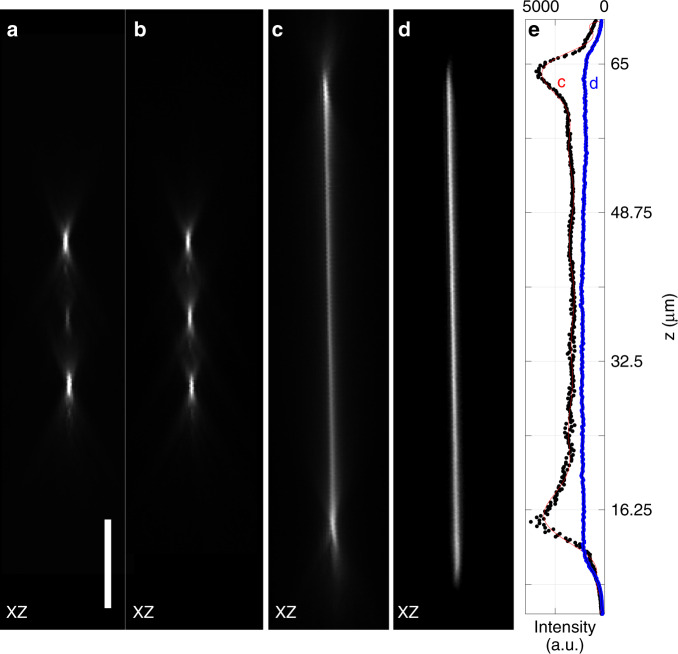
Resonant axial scanning. **a** PSFs obtained using a remote mirror with three steps and a sinusoidal scan motion generated by a resonant galvanometer mirror driven at 12 kHz. **b** Scanning over the same step mirror with a sinusoidal scan motion whose amplitude was adjusted to yield evenly illuminated laser foci. **c** Sinusoidal scan motion over a planar mirror inclined by 7.5°. **d** Same arrangement as in **c**, but with a threefold increased scanning amplitude and a mask to truncate the scan range optically. **e** Axial intensity profiles of the beams shown in **c** and **d**. *XZ* maximum intensity projections of transmission data are shown in **a**–**d**. Scale bar: 10 µm

To demonstrate that our focusing strategies are compatible with faster scanning technologies, we replaced the GSM with a 12 kHz resonant galvanometric mirror. When we scan resonantly over the step mirror with a range spanning the three steps equally, three axial foci are generated, where the middle focus is much dimmer than the outer foci (Fig. 3a). This finding is expected, as the resonant scan spends less time per cycle on the central step than on the outer steps. By lowering the scan amplitude, the intensity of the three focal spots can be equilibrated (Fig. 3b and Supplementary Fig. S10). Balancing the intensity of the three foci is a passive process; i.e. it does not require any laser intensity modulation that is tightly synchronized to the resonant scan motion.

If we scan over a 7.5° inclined planar mirror, the resonant scanner produces an axial line scan with a typical temporally averaged intensity profile for sinusoidal motion (Fig. 3c). In addition, we can normalize the axial intensity distribution without any synchronized laser modulation: we increased the scan range of the resonant galvanometer threefold, such that the approximately linear portion of the sinusoidal oscillation covered the same range as shown in Fig. 3c. We placed a mask in the image space of the 4F telescope between the GSM and OBJ1, which restricted the transmitted laser beam to a smaller lateral-scan range. As the lateral and axial scan ranges are coupled, this allowed us to truncate the axial scan range to a smaller, more homogeneous region at the expense of a lowered temporal duty cycle for illumination (Fig. 3d).

**Fig. 4:**
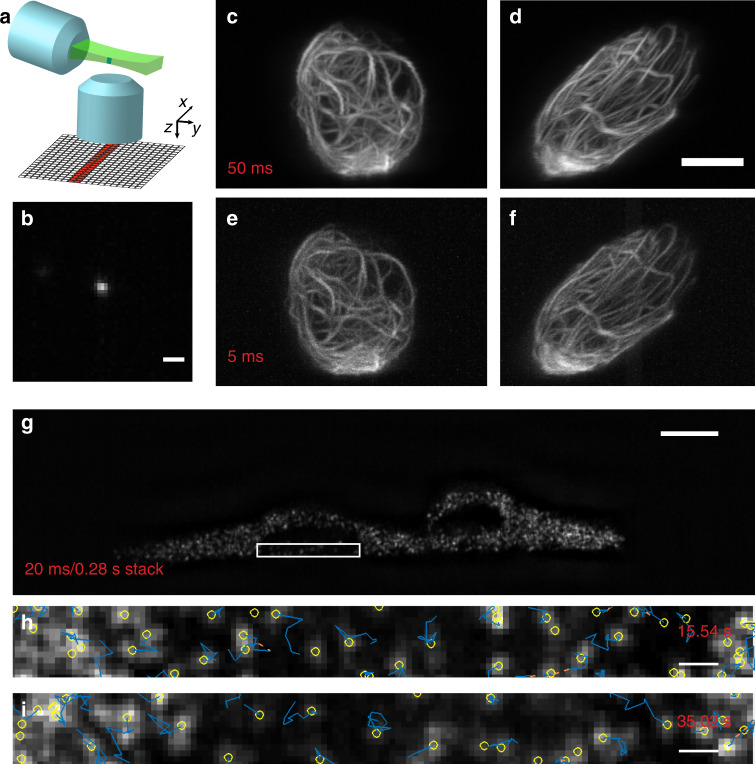
Accelerating axially swept light-sheet microscopy (ASLM). **a** Schematic principle of ASLM: a thin light sheet is scanned in its propagation direction, and only the region within the beam waist (red and green bars) is read out by an sCMOS camera. **b** Point spread function in the *XZ* plane. **c**, **d** RPE cells labelled with GFP-vimentin, imaged with ASLM with a 50 ms integration time. *XZ* and *YZ* maximum intensity projections are shown. **e**, **f** The same cell imaged with ASLM at 5 ms integration time. *XZ* and *YZ* maximum intensity projections are shown. **g** Genetically encoded multimeric nanoparticles (GEMs) inside two MV3 cells, as imaged by ASLM over a 20 ms image integration time, and 3.57 volumes per second. **h**, **i** Axial *YZ* view of the perinuclear region at two timepoints. Yellow circles indicate detected vesicles, and blue lines illustrate cumulative tracks. Scale bars: **b**, **h**, and **i** 1 µm; **d** and **g** 10 µm

To demonstrate the usefulness of our scanning technology to biological imaging, we employed our setup to perform rapid ASLM using a normal, non-resonant galvanometer mirror (Fig. 4a). To this end, a cylindrical lens was used to shape the input beam into a light sheet, which was then imaged into the image space of OBJ1. Using a planar mirror tilted by 7.5° allowed us to scan a thin light sheet (effective NA of 0.8) over a ~40 µm range. The microscope achieves a spatial resolution of 387.3 ± 31.2 and 437.8 ± 42.5 nm in the lateral and axial directions, respectively (mean ± standard deviation, *n* = 15), as evidenced by the PSF (Fig. 4b). We imaged RPE cells labelled with vimentin-GFP at both 50 and 5 ms exposure times per frame. As seen in Fig. 4c–f, the morphological details are faithfully recovered at the ten times faster acquisition time. These results represent a 20-fold improvement over previously reported frame rates for ASLM^20,21^. To leverage this rapid ASLM system, we imaged genetically encoded multimeric nanoparticles (GEMs)^22^ inside MV3 cells. Such imaging has previously only been performed two-dimensionally due to restricted volumetric acquisition speeds^22^. With our system, we can acquire volumetric data (encompassing 13 individual *Z*-planes) at a volumetric rate of 3.5 Hz and isotropic ~400-nm resolution over a volume spanning 128 × 32 × 2 µm^3^ and encompassing 200 timepoints. This volumetric imaging speed was sufficient to track GEMs as they rapidly diffused through the cellular cytoplasm (Fig. 4h–i, Supplementary Fig. S11, Supplementary Video 2). Analysis of the tracks suggests that most particles diffuse with maximum and average diffusion coefficients of 48.9 and 16.3 nm^2^ s^−1^, respectively. Nonetheless, owing to their stochastic Brownian motion, many particles displayed short bursts of rapid translocation with instantaneous diffusion coefficients greater than 815 nm^2^ s^−1^.

Finally, we incorporated our remote-focusing technique into a two-photon raster-scanning microscope to demonstrate its potential for intravital imaging. To this end, a new remote-focusing arm was optimized for near-infrared transmission and was matched to an existing two-photon raster-scanning microscope. The remote-focusing system was designed to utilize a water dipping lens with a numerical aperture of 1.05 to maximize the spatial resolution (‘Methods’, Supplementary Fig. S12). Using a planar gold mirror tilted by 5°, an axial scan range of 55 µm was achieved. To align and characterize the setup, we first installed a conventional galvanometric mirror and measured the PSFs using 200 nm fluorescent nanospheres at different *Z*-positions. We found that the system had close to a diffraction-limited performance (Supplementary Fig. S13) over its full scan range (the lateral resolution varied from 386–437 nm, and the axial resolution varied from 1.7–1.8 µm).

**Fig. 5:**
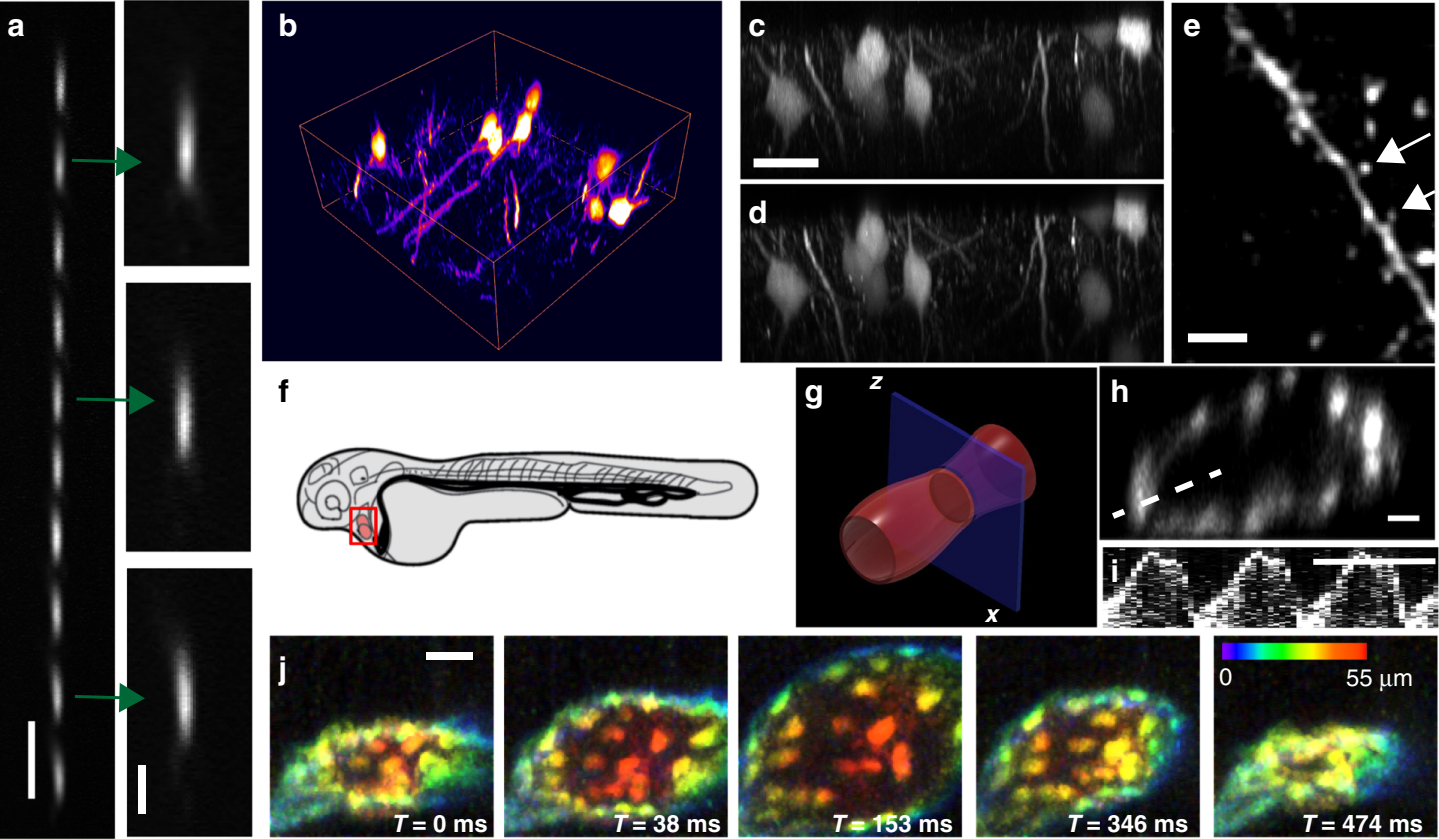
Two-photon microscopy with resonant axial remote focusing. **a** Point spread function using 200 nm fluorescent nanospheres moved to different axial positions, acquired via resonant (12 kHz) remote focusing with a tilted planar mirror (5° tilt). **b** Rendering of a fixed brain slice labelled with Thy1-GFP acquired using resonant remote focusing. **c** Axial *XZ* view of the brain slice shown in **b** using remote focusing. **d** Axial *XZ* view of the same brain slice but acquired with conventional *Z*-stepping. **e** Lateral *XY* view of the brain slice shown in **b**, acquired with resonant remote focusing. Arrows mark individual spines. The image was convolved with a Gaussian filter (sigma = 1 pixel) to suppress spurious noise. **f** Schematic drawing of a zebrafish embryo. **g** Zoomed-in view of the zebrafish heart. The blue plane depicts the axial imaging plane. **h** Averaged (over 30 cycles) *XZ* cross-section of the zebrafish heart labelled with Tg(kdrl:EGFP), acquired with a frame rate of 45 Hz. **i** Kymograph of the beating heart measured along the line shown in **h**. The kymograph uses raw data, and no averaging was applied. **j** Volumetric imaging of the zebrafish heart at a volume rate of 156 Hz, *XY* view with depth encoded in colour. Scale bars: **a** and **e**: 5 µm; **a** (inset): 2 µm; **c**, **h**, and **j**: 20 µm; **i**: 0.5 s

Next, we installed a 12 kHz resonant galvanometric mirror to enable fast axial scanning. Figure 5a shows the PSF measurements along the optical axis of the system, which were obtained by moving 200 nm fluorescent nanospheres with a piezoelectric actuator through the entire *Z*-range of the remote-focusing system. The PSFs remained largely aberration free throughout the axial scan range. The lateral resolution was 0.41 ± 0.05 µm, and the axial resolution was 2.07 ± 0.07 µm (mean ± standard deviation, *n* = 50).

Next, we evaluated our ability to perform volumetric imaging with resonant remote focusing on a fixed brain slice with Thy1-GFP-labelled neurons. Here, *XZ* image planes were acquired with the resonant remote focus in the *Z*-direction and a lateral galvanometer scan in the *X*-direction at a rate of 45 frames per second. After each image plane, a third galvanometer was stepped in the *Y*-direction, which allowed a volumetric image to be acquired. Figure 5b shows a rendering of a volume spanning 55 × 130 × 130 µm^3^ in the *Z*-, *X*-, and *Y*-directions, respectively. To obtain a high image quality, we applied ten times frame averaging, which resulted in an overall three-dimensional stack acquisition time of 114 s. Figure 5c, d shows cross-sectional *XZ* views of the data obtained by remote focusing and a conventionally acquired image dataset (e.g. by *Z*-stepping the sample instead of remote focusing), respectively. Strikingly, the two images are nearly identical, demonstrating the performance of our remote-focusing technique for conventional two-photon microscopy. Indeed, since the high lateral resolution is maintained, fine features, including synaptic spines, can be observed in the *XY* view of the data acquired via remote focusing (Fig. 5e).

To demonstrate the potential of our approach for fast intravital imaging, we imaged the beating heart of 2.5-day-old early zebrafish embryo^23,24^ (schematically shown in Fig. 5f, g) labelled with the vasculature marker Tg(kdrl:EGFP)^25^ at an excitation wavelength of 920 nm (15 mW laser power). By 2.5 days, the looped heart of the embryo is located in the pericardial cavity and is divided into two chambers, the atrium and ventricle, separated by the atrioventricular canal^24^. Due to its fast contractions, the heart has become the holy grail of fast imaging methods, and we report for the first time raster-scanning imaging of the heart in an axial *XZ* imaging plane (Fig. 5h and Supplementary Fig. S14), yielding a cross-sectional view of the heart. Our imaging frame rate of this axial plane amounted to 45 frames per second, which was sufficient to temporally sample the dynamics of the heart over 1340 timepoints and 65 cycles (Supplementary Video 3). The kymograph (Fig. 5i and the zoomed-in version in Supplementary Fig. S15) clearly showed the expected fast contraction and slower relaxation of the heart (Supplementary Video 3). We further explored volumetric imaging of the heart by stacking multiple *XZ* planes in the *Y*-direction, pursuing two strategies. First, by reducing the *XZ* frame size to 128 × 128 pixels, we obtained a two-dimensional imaging frame rate of 156 Hz, which allowed us to image a volume encompassing 21 slices in the *Y*-direction at 7.4 Hz (Supplementary Video 4). In the second strategy, we exploited the periodic beating of the heart: we recorded a series of *XZ* slices for 1 s at a frame rate of 156 Hz for each *Y*-position. Such *XZ* time series were recorded at 201 *Y*-positions sequentially to cover the heart tube with a length of 100 µm. In a post-processing step, we adjusted the phase of each time series such that all the *Y*-positions were synchronous. Therefore, we could obtain a reconstructed volumetric time series of the heart sampled at 156 Hz (Fig. 5j and Supplementary Video 5). As such, our remote re-focusing method could allow a more systematic evaluation of cardiac hemodynamics in vivo and owing to the extended penetration depth of two-photon microscopy, allow this evaluation in older zebrafish.

## Discussion

We present a novel approach to remote focusing that leverages lateral scanning technologies to achieve rapid axial re-focusing. The proposed method is compatible with even faster scanning technologies than that presented here, and it should be possible to reach MHz scan rates. The scanning motion can be resonant or deterministic (i.e. having the ability to freely choose the scanning waveform and to perform discrete scanning steps), depending on the choice of scanning technology. This characteristic contrasts with resonantly driven ETLs, which can only provide sinusoidal waveforms. In addition, compared to the recently published reverberation microscopy, our technique can be utilized to (a) access many more *Z*-positions with greater flexibility, (b) provide scanning free of spherical aberrations, and (c) deliver tailored laser power to each focal plane. However, reverberation microscopy achieves much faster re-focusing than the technology presented here and can also more readily cover a larger axial scan range.

Importantly, neither ETLs nor reverberation microscopy can avoid deleterious spherical aberrations along their axial scan range and hence are not typically employed for high-resolution imaging (Supplementary Fig. S16). In contrast, our technique employs remote focusing in an aberration-free format, which avoids spherical aberrations over its scan range, and hence achieves a more uniform and higher spatial resolution throughout the imaging volume. We have demonstrated in this manuscript that we can indeed resolve fine structural features, including dendritic spines.

The two presented implementations, the step mirror and tilted planar mirror, have different strengths and weaknesses. The step mirror allows arbitrary scan step sizes, but the field of view of the remote scanning system will put an upper limit on the number of steps that can be realized. Our concept can be extended to two scan dimensions in the remote-focusing arm, allowing additional steps to be added in a second lateral dimension (Supplementary Fig. S17). The tilted mirror geometry allows fine axial step sizes that are, in principle, only limited by the angular resolving power of the lateral-scan technology being used. The axial range is tied to the tilt angle and the field of view. Both applications presented in this manuscript do not readily achieve the axial scan range that aberration-free remote focusing can provide using conventional actuators (up to a 200–300 µm scan range for an NA of 0.7–0.8, as previously reported)^2,3,20^. An increased scan range would be especially beneficial for two-photon raster scanning. In contrast, we think that very rapid ASLM will be limited to shorter axial scan ranges, as the shortening of the effective pixel dwell time may become limiting. Our scan range in this publication was limited to 55 µm, which was mainly caused by the small field of view of our scan optics and limited scan amplitude of the galvanometric mirror. Lenses for wide fields (26 mm) have become available and, together with mirror scanners tuned for larger amplitudes, should increase the scan range threefold (see the discussion on the range in Supplementary Note 2). Another way to improve the scan range while maintaining the continuous scanning ability is to employ a two-dimensional lateral scan with a mirror that is tilted in one dimension and has discrete steps in the other dimension (Supplementary Fig. S18). We note that some aberrations are introduced to the axial foci when scanning close to the edges of the field of view of the remote-focusing arm. We anticipate that these aberrations can be minimized by employing telecentric optical components with a large field of view.

Our first practical demonstration of microscopic imaging was ASLM, which has been criticized for its slow acquisition speed (a frame rate of approximately 10 Hz in high-resolution implementations, as reported previously)^20,21^. Here, we demonstrate that our new scanning technology allows one order of magnitude acceleration while maintaining the high spatial resolving power of this emerging imaging technology. ASLM has been implemented with ETLs, but only using thick light sheets using low numerical apertures for excitation^26^. For applications exceeding numerical apertures of 0.3 for light-sheet generation, aberration-free remote focusing is necessary to avoid the deleterious effects of spherical aberrations^20^.

In a second application, we implemented our axial scanning technology in a two-photon raster-scanning microscope and performed high-resolution volumetric imaging with an axial scan rate of 12 kHz. Indeed, at this spatial resolution, our approach is six times faster than previously reported aberration-free focusing technologies, including the high-speed Lissajous scan trajectories presented by Botcherby et al.^2,3^. We then demonstrated the potential of our technology for intravital microscopy by imaging the beating heart of a zebrafish embryo in an *XZ* ‘en face’ geometry at 156 fps. We believe that this opens up major applications for intravital imaging, especially in the neurosciences. Both the discrete and continuous scanning technologies presented here may be used in many applications to image different layers of the brain nearly simultaneously or to rapidly acquire whole volumes to measure neuronal firing patterns or cerebral blood flow. Importantly, unlike previous technologies, our approach is fully compatible with acousto-optical deflectors and thus theoretically capable of axial scanning on the sub-microsecond timescale (e.g. >1 MHz). Thus, using resonant Lissajous scanning patterns^27^, we foresee the possibility for volumetric imaging at kHz rates.

## Materials and methods

### ASLM setup

Laser light emitted from a diode-pumped solid-state 488 nm CW laser (Sapphire 488–500 LPX, Coherent, Inc. Santa Clara, CA, USA) was spatially filtered with a pinhole (P30D, Thorlabs, Newton, NJ, USA) and expanded ninefold (AC254-050-A, AC254-150-A, and GBE03-A, Thorlabs, Newton, NJ, USA). A half-wave plate (10RP52-1B, Newport Corporation, Irvine, CA, USA) was used to rotate the polarization of the beam and vary the laser power. In the remote-focusing arm, we used a 60 × 0.7 NA air objective (CFI S Plan Fluor ELWD 60XC, Nikon Instruments, Melville, NY, USA), two *f* = 200 mm telecentric tube lenses (AC508-200-A, Thorlabs, Newton, NJ, USA), a quarter-wave plate (10RP54-1B, Newport Corporation, Irvine, CA, USA) and a galvanometer scanner (CRS12kHz, Cambridge Technology, Bedford, MA, USA). As this air objective requires a coverslip, a 170-µm-thick coverslip was glued to the front surface of the lens. The illumination arm consisted of a ×40 NA 0.8 water dipping objective (CFI Apo NIR 40X W, Nikon instruments, Melville, NY, USA) and two achromatic doublets (AC508-150-A and AC508-300-A, Thorlabs, Newton, NJ, USA). A custom-made sample chamber was used into which the water dipping lens was immersed. The relay lenses were chosen such that the overall demagnification from the remote mirror to the water chamber corresponds to 1.333. A traditional light-sheet detection arm was oriented at 90° (see also Fig. 1) and consisted of a ×40 NA 0.8 water dipping objective (CFI Apo NIR 40X W, Nikon Instruments, Melville, NY, USA), a *f* = 200 mm telecentric tube lens (TTL200-A, Thorlabs, Newton, NJ, USA) and a scientific CMOS camera (Orca Flash 4.0, Hamamatsu Photonics, Hamamatsu City, Japan). For fluorescence detection, a long-pass filter (BLP01-488R, Semrock, Rochester, NY, USA) was used to block the laser light, and the sample was scanned through the ASLM illumination beam with a piezo stage (PIHera, P-621.1CD, Physik Instrumente, Karlsruhe, Germany). To evaluate the beam in transmission, another detection arm was built with neutral density filters (ND40A, Thorlabs, Newton, NJ, USA) to attenuate the laser. An objective piezo stage (PIFOC, Physik Instrumente, Karlsruhe, Germany) was used to acquire three-dimensional stacks of the laser spots using *Z*-stepping of the objective (i.e. the tertiary objective corresponding to the imaging arm). For the fluorescence detection arm, no provisions for *Z*-stepping of the detection objective were made.

### Two-photon raster-scanning microscope setup

The setup of the two-photon raster-scanning microscope is illustrated in Supplementary Fig. S12. A Ti:sapphire laser (Coherent Vision-S, Coherent, Inc. Santa Clara, CA, USA) tuned to 900 nm with a maximum output power of 2.4 W (100 fs pulse width, 80 MHz repetition rate) was used for two-photon excitation. The laser power was controlled with a Pockels Cell (350-80-02, Conoptics, Danbury, CT, USA) spatially filtered with a pinhole (PH-100, Newport Corporation, Irvine, CA, USA) and expanded fourfold by a Galilean telescope (AC254-50-B, AC254-200-B, Thorlabs, Newton, NJ, USA). A half-wave plate (AHWP05M-980, Thorlabs, Newton, NJ, USA) was used to adjust the maximum laser intensity reflected by the polarizing beam splitter cube (PBS252, Thorlabs, Newton, NJ, USA). In the remote-focusing arm, we used a 20 × 0.8 NA air objective (UPLXAPO, Olympus Scientific Solutions, Waltham, MA, USA), a tube lens (TTL200MP2, Thorlabs, Newton, NJ, USA), a scan lens (LSM54-850, Thorlabs, Newton, NJ, USA), a quarter-wave plate (AQWP6, Bolder Vision Optik, Boulder, CO, USA), and a galvanometer scanner (CRS12kHz, Cambridge Technology, Bedford, MA, USA). As this air objective requires a coverslip, a 170-µm-thick coverslip was glued to the front surface of the lens. For the remote mirror, we selected a protected gold mirror for its high damage threshold of 2 J/cm^2^ (PFSQ10-03-M01, Thorlabs, Newton, NJ, USA). The illumination arm consisted of a ×25 NA 1.05 water dipping objective (XLPLN25XWMP2, Olympus Scientific Solutions, Waltham, MA, USA), a scan lens (SL50-2P2, Thorlabs, Newton, NJ, USA), a tube lens (TL200-2P2, Thorlabs, Newton, NJ, USA), two F-theta lenses (S4LFT0075, Sill Optics, Wendelstein, Germany), and two galvanometer scanners (6215H, Cambridge Technology, Bedford, MA, USA). The relay lenses (AC254-250-B, Thorlabs, Newton, NJ, USA) connected the remote-focusing arm and the illumination arm. The overall demagnification from the remote objective to the primary objective corresponded to 1.35. Fluorescence was collected through the same objective, reflected by a dichroic mirror (FF735-Di02-50.8-D, Semrock, Rochester, NY, USA), filtered by a short-pass filter (FF02-694/sp-25, Semrock, Rochester, NY, USA) and a bandpass filter (FF01-527/70-25, Semrock), and focused with an achromatic doublet lens (AC254-45-A, Thorlabs, Newton, NJ, USA) onto a PMT (H7422-40, Hamamatsu Photonics, Hamamatsu City, Japan). The signals from the PMT were amplified and filtered with an amplifier (DLPCA-100, Femto, Berlin, Germany) and then digitized with an FPGA (NI PXIe-7961R and NI 5734 DAQ, National Instruments, Austin, TX, USA). A three-axis piezo sample stage (Nano-3D200, Mad City Labs Inc., Madison, WI, USA) was used to move the sample precisely. The microscope was controlled using ScanImage (Vidrio Technologies, LLC, VA, USA). *XZ* plane images were typically acquired with a 45-Hz frame rate and a 90% spatial fill fraction for an image size of 512 × 512 pixels and 156-Hz for an image size of 128 × 128 pixels. *XY* plane images were acquired with an image size of 512 × 512 pixels and a 1 Hz frame rate. For the high-resolution structural images, all the stacks are averages of ten consecutive frames. The average power used under the objective was ~3 mW for the fixed mouse brain slice and ~15 mW for the zebrafish.

### Focal spot measurements

To measure the optical quality of our remote-focusing technique, we acquired transmission datasets with the tertiary imaging system shown in Fig. 1. To this end, the tertiary imaging objective was carefully aligned along the optical axis of OBJ2, and an image of the laser spot was formed on the camera. To acquire three-dimensional data, we stepped the tertiary objective with the objective piezo. The step size was chosen to be 160 nm, which results in isotropic voxel sizes, and typically, a volume with a *Z*-range of 100 µm was acquired, which encompasses the full refocus range used in this work. This procedure was repeated for each focal position when re-focusing was performed in discrete steps (i.e. when the regular galvanometric mirror was used in discrete steps). Similarly, when the galvanometer was driven by a triangular waveform or the resonant scanner was used, a three-dimensional transmission stack was acquired. Since resonant scanning is much faster than the acquisition of the three-dimensional stack, a time-averaged intensity distribution of the rapidly moving focus was acquired.

### Fabrication of micro step mirror using UV lithography

Five micrometres of SU-8 3005 was spin-coated (WS-650MZ-23NPPB, Laurell Technologies Corporation, North Wales, PA, USA) at 3000 RPM onto a 3-inch silicon wafer. The wafer was soft baked and treated according to the protocol of the photoresist distributor (Microchem, Round Rock, TX, USA). A microfluidic mask (showing a rectangular pattern/an edge) was projected for 20 s with the setup described^28^ onto the wafer. The wafer was post-baked at 95 °C so that the interfaces between developed and undeveloped regions become visible. Afterwards, the wafer was spin-coated again with 5 µm SU-3005 using the same procedure as above. The wafer was exposed to the same mask a second time, but this time, it was rotated and translated by a few millimetres in one direction compared with the former exposure. In this fashion, a micro-staircase with a continuously varying step width that ranged from millimetres to µm over a wide region of the wafer was fabricated. Translation of the mask in one direction only allows parallel steps to be fabricated. The whole wafer was again baked at 95 °C and finally rinsed with propylene glycol methyl ether acetate and isopropanol. Finally, the wafer was covered with a 100 nm layer of platinum to make the surface reflective using a sputter coater (Q150T ES Turbo-Pumped Sputter Coater/Carbon Coater, Quorum Technologies, East Sussex, UK). A profilometer (DektakXT, Bruker, Tucson, AZ, USA) was used to measure the height of the steps and to optimize the procedure. A reflectance light microscopy image of the mirror is shown in Supplementary Fig. S3. The heights of the step were ~5.34 and 6.73 µm.

### Mammalian cell culture and labelling

The hTERT-immortalized human retinal pigment epithelial cells (hTERT-RPE-1) were obtained from ATCC (Manassas, VA) and grown in DMEM/F12, 1:1 (Gibco, Thermo Fisher Scientific Inc., Waltham, MA, USA) medium supplemented with 10% foetal bovine serum (Gibco, Thermo Fisher Scientific Inc., Waltham, MA, USA), penicillin/streptomycin (Gibco, Thermo Fisher Scientific Inc., Waltham, MA, USA), and 0.01 mg/ml hygromycin-B (Millipore Sigma, Darmstadt, Germany). The cells were tagged with mEmerald-vimentin using the TALEN genome editing approach, which has been previously described^29^. For imaging, the cells were placed into collagen as previously described^21^. MV3 cells were obtained from Peter Friedl (MD Anderson Cancer Center, Houston TX). The MV3 cells were cultured in DMEM (Gibco, Thermo Fisher Scientific Inc., Waltham, MA, USA) supplemented with 10% foetal bovine serum (Thermo Fisher Scientific Inc., Waltham, MA, USA) at 37 °C and 5% CO_2_. The cells were infected to express GEMs using a lentiviral construct from Addgene (Plasmid #116934^22^). The cells were sorted by fluorescence activated cell sorting to purify the population of cells expressing GEMs.

### Animal specimens

All animal protocols were approved by local Institutional Animal Care and Use Committees as directed by the National Institutes of Health and strictly followed. This included APN 2016-101805 (to Dr Stephen Skapek, UT Southwestern Medical Center) and 101715 (to Dr Ilya Bezprozvanny, UT Southwestern Medical Center).

### Zebrafish sample

To demonstrate fast axial scanning on the two-photon microscope, we imaged 2.5-day-old zebrafish labelled with the vascular marker Tg(kdrl:EGFP)^25^. The zebrafish embryos were embedded in 5% methylcellulose on an agarose-coated dish.

### Fixed mouse brain sample

The fixed mouse brain sample was a kind gift from the laboratory of Dr Ilya Bezprozvanny. In short, a Thy1-GFP mouse was transcardially perfused first with cold PBS and then with 4% PFA in PBS as described previously^20^. The brain was isolated and further fixed in 4% PFA in PBS overnight at 4 °C.

### Reconstruction of the zebrafish heart

For the first strategy employed to image the zebrafish heart (i.e. acquiring rapidly a small stack encompassing 21 y slices), no additional post-processing was needed, as the heartbeat was properly sampled over the whole volume. For the second strategy, we imaged at every *Y*-position a time series of 156 *XZ* planes, then we incremented the *Y*-position and acquired another time series of *XZ* planes. As long as the zebrafish does not move or change its heartbeat, a large three-dimensional stack can be built in this way. Each of the *XZ* time series contains more than one heartbeat cycle, but the different slices along the *Y*-direction are not in phase. To align the slices in time to each other, the following procedure was performed: Because there was no additional time interval between *XZ* slices, the last frame of one slice was followed by the first frame of the next slice. Accordingly, we calculated the phase shift of each slice. Therefore, we could reconstruct the volume based on the phase shift and the average period of the heartbeat. We noticed that there were inconsecutive fringes in the reconstructed three-dimensional image during rapid heart systole, which was caused by the variability in the heartbeat period during acquisition. To minimize these fringes, we first projected the three-dimensional images in the *XY* view and then resliced the stacks to show the time dimension in the two-dimensional plane intuitively. After aligning the images in the time dimension, we could obtain the additional shift of the period caused by the variability. With this information, we obtained smooth reconstructed three-dimensional images of the beating heart of the zebrafish, which agrees well with our volumetric imaging at a 7.4 Hz volume rate of the same zebrafish heart.

### Particle detection and analysis

GEMs were detected and tracked using the freely available MATLAB-based uTrack software package^30^ (see https://github.com/DanuserLab). For detection, the ‘Watershed Applegate’ method was used on the difference in the Gaussian filtered data, with high- and low-frequency Gaussian filter sizes of 1 and 5 pixels, respectively. The minimum intensity threshold for the watershed algorithm was identified empirically. For tracking, a Brownian motion model was adopted with a maximum gap closing of one frame and a minimum track length of six frames. Because GEMs are expected to exhibit Brownian motion, no directed motion position propagation was included in the frame-to-frame linking algorithm.

## Supplementary information

Supplementary Material

Supplementary Video 1

Supplementary Video 2

Supplementary Video 3

Supplementary Video 4

Supplementary Video 5

## Data Availability

The datasets acquired for this study are available from the corresponding authors upon request.

## Code availability

The Zemax simulation code is available from the corresponding author upon request. The instrument control software can be requested for academic use from the corresponding author and will be delivered under material transfer agreements with the Howard Hughes Medical Institute and UT Southwestern Medical Center.
