# Supplementary material for: Converting lateral scanning into axial focusing to speed up three-dimensional microscopy
Source: Light Sci Appl. 2020 Sep 18;9:165. doi: 10.1038/s41377-020-00401-9 (PMC7501866; doi:10.1038/s41377-020-00401-9)
Supplement: Supplementary file 1 — Supplementary Material [file 41377_2020_401_MOESM1_ESM.docx]

**Supplementary Information**

Converting lateral scanning into axial focusing to speed up three-dimensional microscopy

**Authors**

Tonmoy Chakraborty,^1^ Bingying Chen,^1^ Stephan Daetwyler,^1^ Bo-Jui Chang,^1^ Oliver Vanderpoorten,^2^ Etai Sapoznik,^1^ Clemens Kaminski,^2^ Tuomas P.J. Knowles,^3^ Kevin M. Dean,^1^ and Reto Fiolka ^1,4^.

**Affiliation**

1 – Department of Cell Biology, UT Southwestern Medical Center, Dallas, TX, USA.

2 – Department of Chemical Engineering and Biotechnology, University of Cambridge, Philippa Fawcett Drive, Cambridge, CB3 0AS, UK

3 – Department of Chemistry, University of Cambridge, Lensfield Road, Cambridge, CB2 1EW, UK.

4 – Lyda Hill Department of Bioinformatics, UT Southwestern Medical Center, Dallas, TX, USA.

**Correspondence**

[Reto.Fiolka@utsouthwestern.edu](mailto:Reto.Fiolka@utsouthwestern.edu)

**Supplementary Notes**

**Note 1, Simulation of the remote focusing setup using Zemax**

This supplementary note describes the results of a simulation of the remote focusing setup with a tilted mirror using Zemax Optic Studio 18.4.1. The simulation comprised of a beam splitter, a galvo-scanning mirror, a remote mirror, four achromatic doublets (Zemax model of AC508-200-A, Thorlabs) for two 4f systems, and two water immersion 40x NA 0.8 objective models which were kindly provided by Ryan McGorty^1^ (Supplementary Figure S1). It has to be noted that in our experimental setup, we use an air objective as the remote objective, which however was not available to us as a model for simulations. To find the right distances (thicknesses) of the optical surfaces in the simulation, we applied optimization functions available in Zemax such as “Quick Adjust” and the “Merit Function Editor” and manually changed parameters using the Slider adjustment in the optimization tab of Zemax.

In the simulation, an incoming collimated beam with defined aperture was reflected 90° degrees by a mirror onto a galvo-scanning mirror tilted by 45°± 1.5° degrees corresponding to ± 3° optical scan angle. The galvo-scanning mirror was conjugated to the back focal plane of a remote focusing objective (40x, NA 0.8) with a telescope of two achromatic doublets of two-inch diameter with f=200 mm (AC508-200-A, Thorlabs). The remote focusing objective focused the beam on a remote mirror which was tilted by 2.5°, 5° or 7.5° degrees (Supplementary Figure S2). To ensure that the incoming beam was reflected onto the same beam path, we optimized the chief ray to be reflected exactly onto the incoming beam by adding an off-axis shift *d* to the objective. The reflected beam travelled back through the above system (objective and 4f system) and was descanned by the galvo-mirror. Then, the galvo-mirror was conjugated with another 4f-system to the back focal plane of the imaging objective. Scanning the beam in the remote focus objective over the tilted mirror, therefore scanned the beam position along the imaging axis. For each mirror tilt (2.5°, 5°, 7.5° degrees), we determined the final focus position manually for 7 galvo-mirror scan positions (-2°, -1.5°, -1°, 0°, 1°, 1.5°, 2° scan mirror tilt). To the obtained values, we fitted a 4^th^ degree polynomial (Supplementary Figure S3A) to determine for each mirror tilt the corresponding focus position within the scan range of the imaging objective. This enabled generating automatic measurements of the PSF and their width as well as the Strehl ratio along the scan direction using Zemax’s inherent ZPL programming language (Supplementary Figures S3-4). Simulation results showed that the PSF in y-direction of the laser foci along the scanning direction in the imaging space was skewed (Supplementary Figure S3F). By manually adjusting the off-axis shift *d* of the objective, this tilt could be straightened (Supplementary Figure S4F) at the cost of a small decrease of resolution (Supplementary Figure S4).

**Note 2: Axial scan range using a tilted mirror**

The change of focus dz that can be achieved with a tilted mirror is given:

$$dz=2\cdot x\cdot\sin\theta\cdot\frac{n1}{n2}$$

Where θ is the tilt angle of the mirror, x is the lateral displacement of the laser focus, n1 is the refractive index of the immersion media of the remote objective and n2 is the refractive index of the immersion media of Objective 2. A graphical schematic is shown in supplementary Figure S5, which shows the geometry giving rise to the sine projection of the lateral scan, and the doubling of this displacement by the mirror. Lastly, the factor n1/n2 arises from the required magnification between the remote objective and primary objective to fulfill the aberration free focusing condition.

Here we discuss the implications of this relationship on the axial scan range in both of our setups. In our setup used for the transmission focus measurements and the fast ASLM, we used a 60X objective and the tube lenses limited us to a FOV of 20 mm, which equals to a scan range over the remote mirror of ~333microns. According to the above formula, this corresponds to a maximum axial scan range of 65 microns with a tilt angle of 7.5 degrees. In practice, we noted that at the end of the scan range, field curvature imposed a notable tilt onto the final focus and a more practical linear range was around ~40 microns.

The tilt angle itself is limited by the remote objective: in order to use the full NA of objective 2 for focusing, the remote objective needs to possess a larger angular aperture than Objective 2. In our experimental example, the half opening angle of the NA 0.7 air objective is 44.4 degrees and the half angle of Objective 2 (NA 0.8, water) is 36.9 degrees, which allows us to tilt the beam by ~7.5 degrees without losing any numerical aperture of the imaging system.

There are higher NA air objectives (as high as NA 0.95) and hence potentially larger tilt angles could be used to increase the axial scan range. However, we believe a better strategy is to keep the tilt angle small and instead increase the field of view of the remote objective.

Indeed, recently a 20X NA 0.8 air objectives and matching tube lenses with a field of view of 26.5mm have been developed (e.g. Olympus UPLXAPO20X and SWTLU-C). Building a remote focusing arm with these components and using a scan range of 1.325 mm and a tilt of 7.5 degrees would enable an axial scan range of 260 microns. From our experience, the practical range (linear step response, no curving) is estimated to be smaller, on the range of 150 microns (60% of predicted full range). Some methods to use more of the available scan range could be envisioned, like optical components to correct the optical tilt and nonlinear waveforms driving the galvanometric mirror to linearize the axial response (when non-resonant galvanometric mirrors are used).

In our setup for the 2-photon microscopy, we used a 20X NA 0.8 objective as the remote and a NA 1.05 25X objective for imaging. In this configuration, the half opening angle is only a little bit larger for the remote objective (53.1 degrees) compared to the primary objective (52 degree). Therefore, excessive tilting of the remote mirror, and corresponding lateral adjustment of the remote objective, will reduce the effective NA of the overall system in one direction. For this reason, we settled on a more moderate tilt angle of 5 degrees. This reduces the NA of this microscope system to 1 in one direction and leaves it at NA 1.05 in the other direction, which in theory should only cause a modest resolution loss in one dimension.

With the scan range of the resonant galvo (10 degrees peak to peak), this resulted in a lateral scan range of 425 microns over the remote mirror. With a mirror tilt angle of 5 degrees, this corresponds to an axial scan range of 56 microns, which is in excellent agreement with our experimental observations (55 microns).

The used remote objective lens supports a field of view of 1.325mm, which is three times larger. However, in our system, the available scan lens and tube lens do not support the necessary 26.5mm FOV in image space, and the resonant galvo did not have sufficient scanning amplitude.

Nevertheless, with specialized tube and scan lenses, and higher amplitude scanning mirrors, a threefold increased axial scan range of 173 microns should be possible. Estimating the practical range (i.e. only straight portion of the scan) as 60% of this, this would result in a 100 microns range.

**Note 3: Geometrical considerations for the step sizes of the mirror**

Here we analyze the relationship of the step size of the mirror in relation to its height and the half opening angle of the laser focus, using geometrical optics as an approximation. In Supplementary Figure S6 it can be seen that the minimal width dx for the n-th step with height dz and the half opening angle α scales in the in the following:

$dx=\left|\left(2n+1\right)\cdot dz\cdot\tan\alpha\right|\text{ where, }n=0,\text{ }\pm1,\text{ }\pm2,...$

Here n=0 correspond to the nominal focal plane. Obviously increasingly wider steps have to be used with increasing defocus. To give the reader an idea how many steps can be realistically fit into a field of view, we provide a few examples, where we numerically filled increasingly wider steps into a fixed interval.

For our setup, a half opening angle of α=36 degrees and a field of view / scan range of 330 microns is assumed for our remote focusing objective. To achieve 1 microns z-steps in the primary objective, a mirror step size dz of 0.6666 microns is required. Using the above relationship for the width of the step sizes, we found that one can fit 26 steps into this field of view (i.e. the axial scan range would be 26 microns in 26 axial steps). Using recently developed large Field number objectives and tube lenses (e.g. UPLXAPO20X and SWTLU-C), a FOV of 1.325mm is possible, which would result in 52 steps.

**Supplementary Figures**


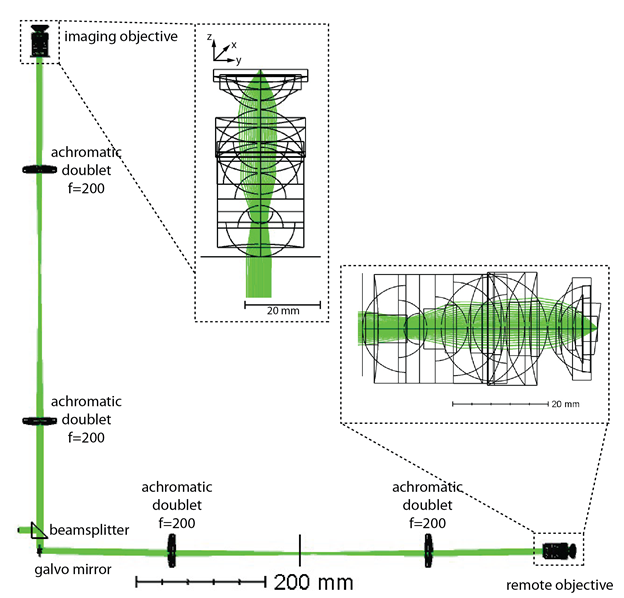


**Supplementary Figure S1 Schematic overview of the simulation.** Renderings from Zemax 3D viewer as overview about the simulation (scale bar 200 mm). Inlets highlight the remote objective and the imaging objective (scale bar 20 mm). Here, a galvo-mirror scan angle of 1,152° degrees is displayed. The incoming beam was set at an aperture of 6.43 mm, and the mirror tilt was set to 7.5° degrees.


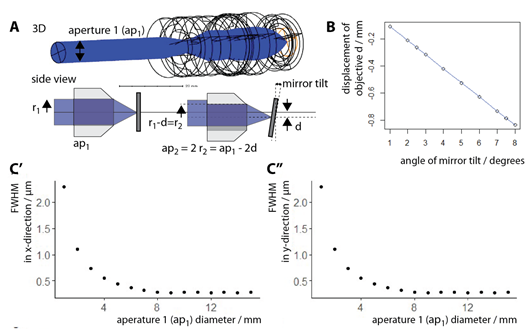


**Supplementary Figure S2 Determination of aperture of incoming beam.** **(A)** Schematic drawing of dependence of the maximal available aperture of the imaging system in relation to the mirror tilt (*top*: aperture ap_1_ in case of straight mirror, *bottom*: schematic comparison of straight vs tilted mirror). To ensure that the outcoming beam was reflected on the same optical path as the incoming beam, the objective was displaced from the central optical axis. Objective displacement (d), however, reduced the maximal available aperture (ap_2_ with radius r_2_) in relation to the aperture of the system with a straight mirror (ap_1_ with radius r_1_) according to the relation ap_1_ – 2d. Therefore, to determine the available aperture for a given mirror tilt, the required displacement (d) and the unperturbed aperture (ap_1_) had to be determined for the given objective. **(B)** We measured the required displacement of the objective d in relation to the mirror tilt by overlapping the chief ray of the outcoming beam (after the mirror) onto the chief ray of the incoming beam (black dots). The dependence was linear (blue line, R-squared =1). **(C’, C’’)** To determine the maximal available aperture of the objective, we determined the FWHM of the PSF in x-direction (C’) and y-direction (C’’). After an aperture of 8 mm, the FWHM did not decrease anymore. Therefore, we set ap_1_ = 8 mm.


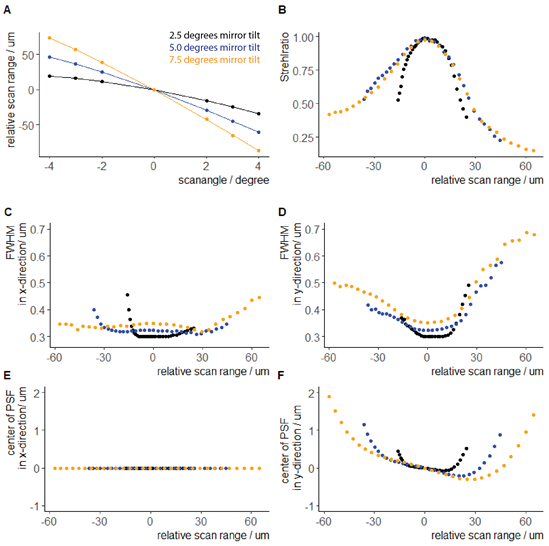


**Supplementary Figure S3 Simulation PSF measurements over scan range**. We measured the properties of the Huygens PSF of the simulated imaging system at the focus of the beam in the imaging chamber at 2.5° (black), 5.0° (blue) and 7.5° (orange) degrees mirror tilt of the remote mirror over a range of 3° optical scan angle of the galvo-mirror. In this setup, the chief ray of the incoming beam was matched onto the chief ray of the reflected beam at the mirror. **(A)** Dependence of the relative scan range on the galvo-mirror scan angle. We measured the values at -2°, -1.5°, -1°, 0°, 1°, 1.5°, 2° scan mirror tilt (points) and fitted a 4^th^ degree polynomial to the values (line). **(B)** Changes of the Strehlratio over the relative scan range. **(C)** Full width half maximum (FWHM) measured at the center of the PSF in x-direction. **(D)** Full width half maximum (FWHM) measured at the center of the PSF in y-direction. **(E)** Shift in the center of the Huygens PSF in x-direction over the simulated scan range. **(F)** Shift in the center of the Huygens PSF in y-direction over the simulated scan range.


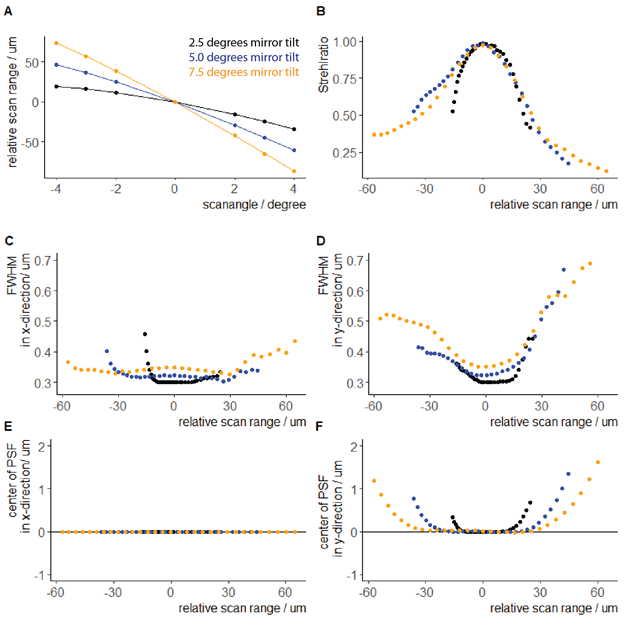


**Supplementary Figure S4 Simulation PSF measurements over scan range in optimized setup.** We measured the properties of the Huygens PSF of the simulated imaging system at the focus of the beam in the imaging chamber at 2.5° (black), 5.0° (blue) and 7.5° (orange) degrees mirror tilt of the remote mirror over a range of ± 3° optical scan angle of the galvo-mirror. In this setup, the objective displacement was optimized to place the focus of the PSF onto the geometric center (x-shift = 0 mm, y-shift=0 mm). **(A)** Dependence of the relative scan range on the galvo-mirror scan angle. We measured the values at -2°, -1.5°, -1°, 0°, 1°, 1.5°, 2° scan mirror tilt (points) and fitted a 4^th^ degree polynomial to the values (line). **(B)** Changes of the Strehlratio over the relative scan range. **(C)** Full width half maximum (FWHM) measured at the center of the PSF in x-direction. **(D)** Full width half maximum (FWHM) measured at the center of the PSF in y-direction. **(E)** Shift in the center of the Huygens PSF in x-direction over the simulated scan range. **(F)** Shift in the center of the Huygens PSF in y-direction over the simulated scan range.


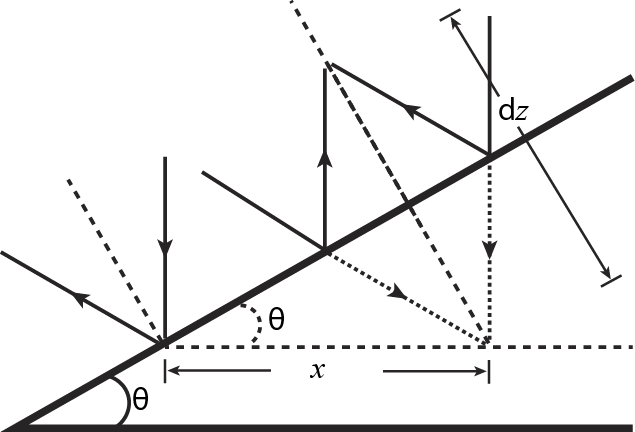


**Supplementary Figure S5** Schematic diagram showing the focus shift dz when scanning over a tilted mirror with a beam tilted such that it is incident normal to the mirror surface.


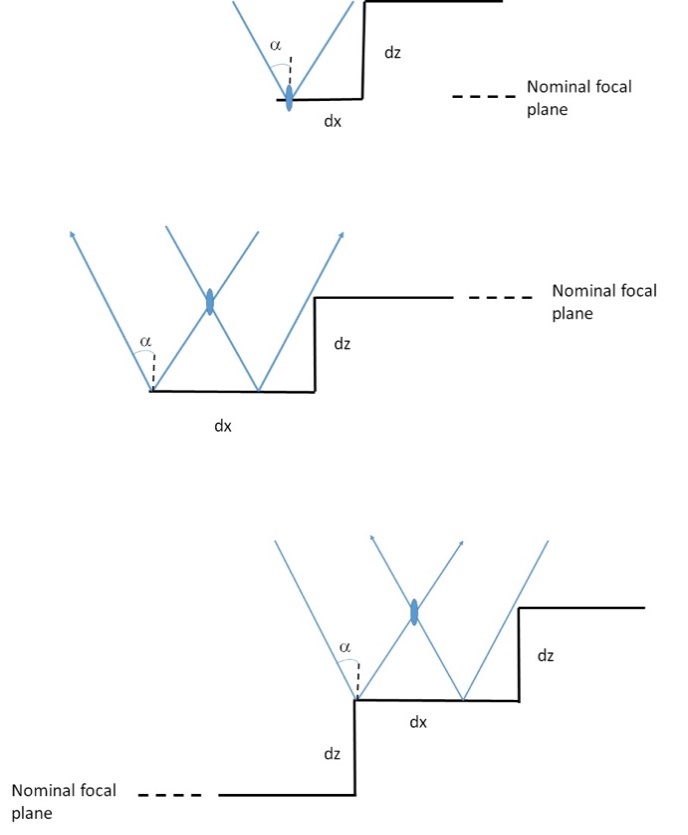


**Supplementary Figure S6** Geometrical considerations for the width (dx) of the mirror steps in relation to the half opening angle Θ and the step height dz. Top graphic shows the scenario where the step is in the nominal focal plane. Middle graphic shows a step that is below the nominal focal plane and bottom graphic shows the scenario where the step is above the nominal plane. Blue lines depict the marginal ray of the laser focus and arrows indicate the propagation direction of the light.


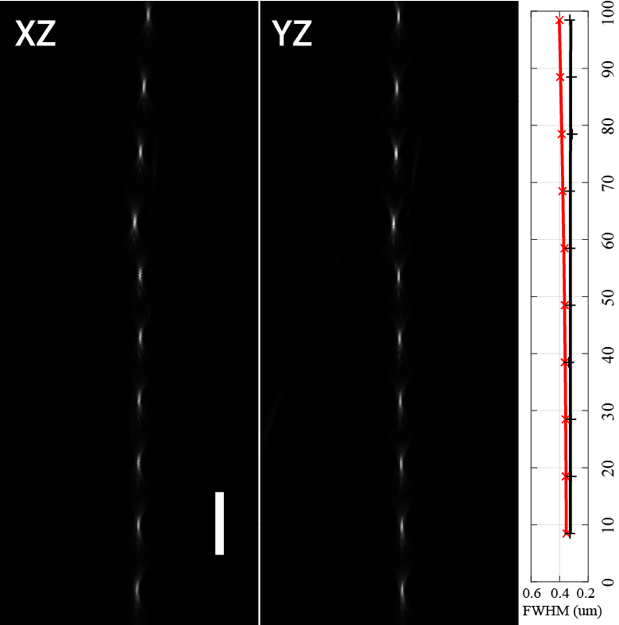


**Supplementary Figure S7** XZ and YZ view of experimental remote focusing using a planar mirror at zero-degree tilt, which was mechanically stepped in the axial direction. This method is expected to be aberration free and serves as a reference for focus quality. For the right-hand-side panel, Red: XZ and Black: YZ. Scale bar: (a-d) 10 microns.


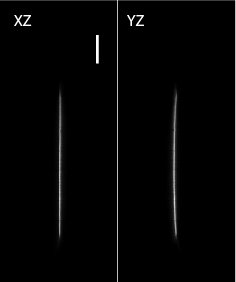


**Supplementary Figure S8** XZ and YZ view of continuous axial scanning obtained using a mirror tilt of 7.5 degree by driving the GSM with a triangular waveform at 100Hz. The YZ view shows the bent in the axial scan when the edges of the field of view of the remote scanning system are reached. Scale bar: (a-d) 10 microns.

**Supplementary Figure S9 Linearity of axial focus position to increments in galvo scanning angle.** Axial focus position is plotted for different control voltages applied to a non-resonant galvanometric mirror. This data was measured on the transmission setup shown in Figure 1, i.e. a remote focusing system with a planar mirror inclined by 7.5 degrees.


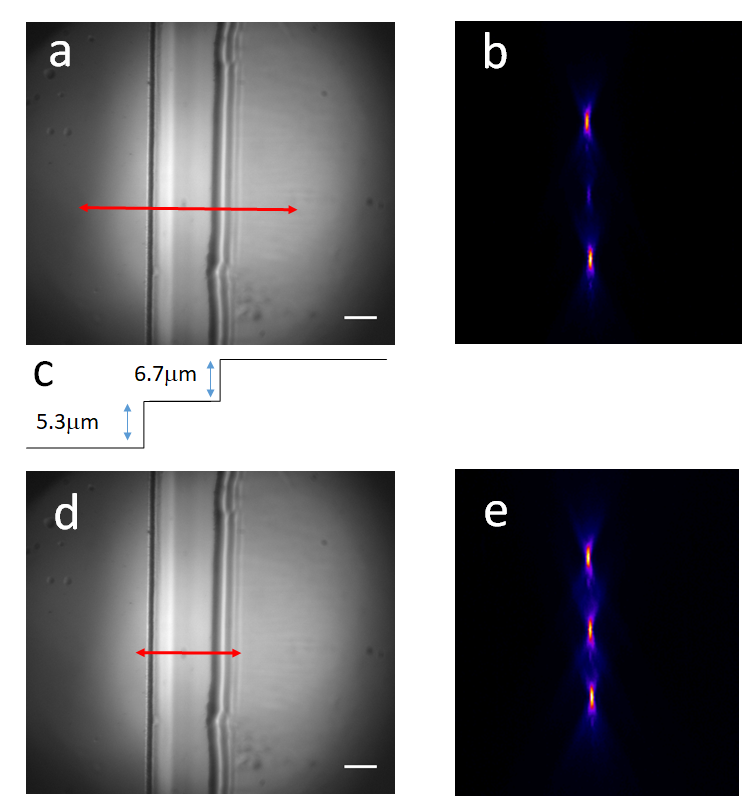


**Supplementary Figure S10 Resonant scanning over a step mirror.** (a) Reflectance image of the step mirror. Red line shows the range of the resonant scan over the mirror. (b) Corresponding axial foci resulting from the scan shown in (a). (c) Schematic height profile of the step mirror shown in (a). (d) Reflectance image of the step mirror. Red line shows the range of the resonant scan with reduced amplitude. (e) Corresponding axial foci resulting from the scan shown in (d). Scale bar: (a,d) 20 microns.


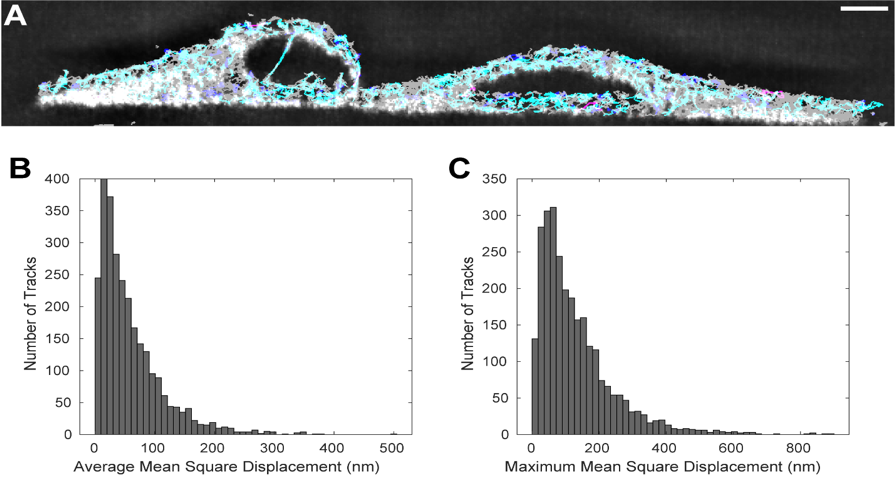


**Supplementary Figure S11** Tracking results of genetically encoded multimeric nanoparticles (GEM) inside two MV3 cell. (a) X-Y Maximum intensity projection over whole imaging volume shown in Figure 3 (h)-(i), showing cumulative tracks. (b) Average Mean Square Displacement. C Maximum Mean Square Displacement.


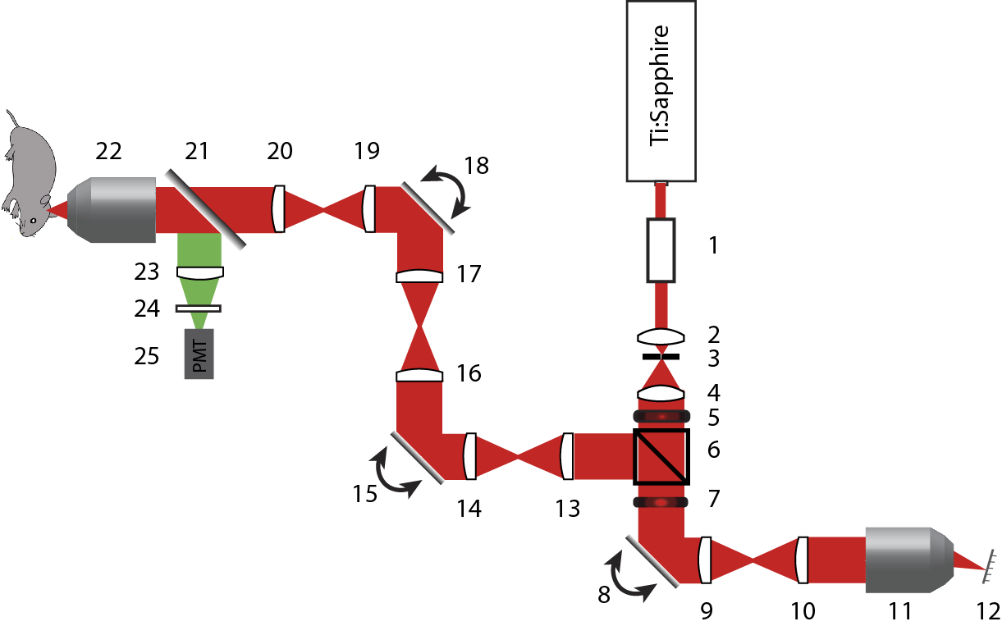


**Supplementary Figure S12** Schematic representation of the 2-photon raster scanning microscope with remote focusing capability. 1, Pockels Cell (350-80-02, Conoptics); 2, AC254-50-B (Thorlabs); 3, pinhole (PH-100, Newport), 4, AC254-200-B (Thorlabs); 5, half-wave plate (AHWP05M-980, Thorlabs); 6, beam splitter cube (PBS252, Thorlabs); 7, quarte-wave plate (AQWP6, Boldervision); 8, Galvo scanner (CRS12kHz, Cambridge Technologies); 9, scan lens (LSM54-850, Thorlabs); 10, tube lens (TTL200MP2, ThorLabs); 11, 20X 0.8 NA air objective (UPLXAPO, Olympus), 12, gold mirror (PFSQ10-03-M01, Thorlabs); 13-14, (AC254-250-B, Thorlabs); 15, Galvo scanner (6215H, Cambridge technology), 16-17, F-theta lenses (S4LFT0075, Silloptics); 18, Galvo scanner (6215H, Cambridge technology); 19, scan lens (SL50-2P2, Thorlabs; 20: tube lens (TL200-2P2, Thorlabs); 21, dichroic mirror (FF735-Di02-50.8-D, Semrock): 22, 25X NA 1.05 water dipping objective (XLPLN25XWMP2, Olympus); 23, AC254-45-A (Thorlabs); 24, a short-pass filter (FF02-694/sp-25, Semrock) and a bandpass filter (FF01-527/70-25, Semrock); 25, PMT (H7422-40, Hammamatsu Inc).


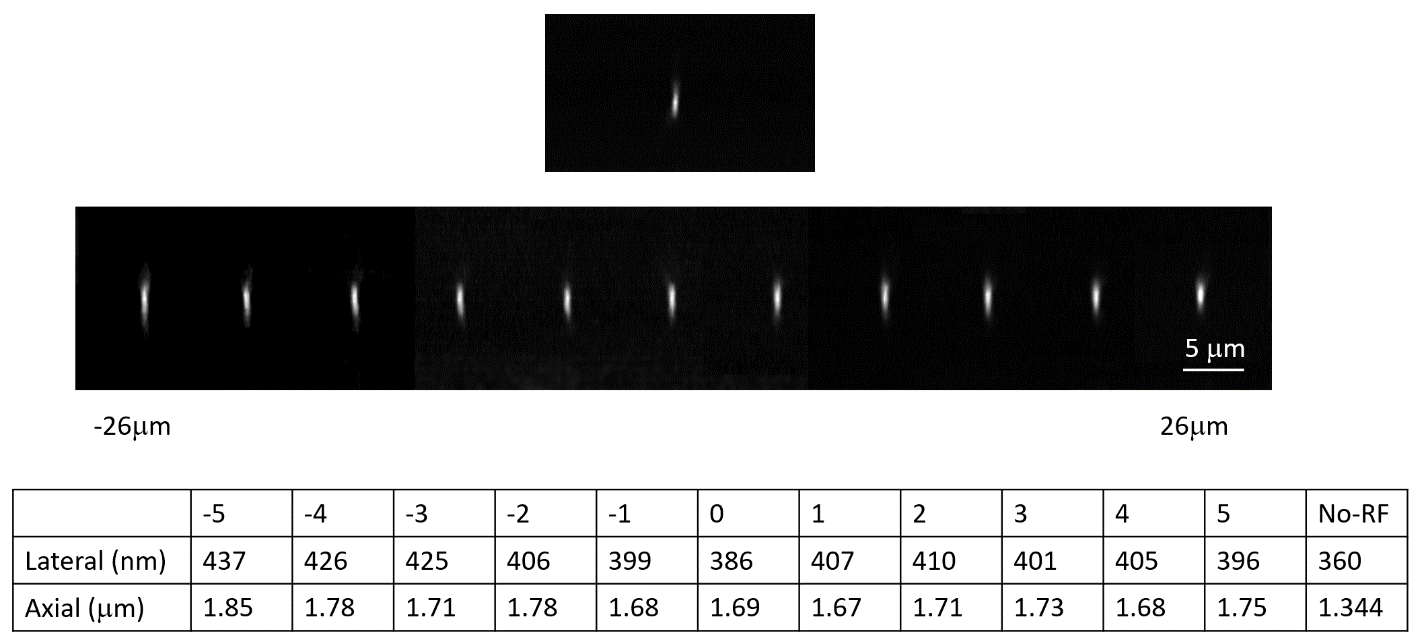


**Supplementary Figure S13 Point spread functions of the 2-photon microscope setup, measured with 200 nm green fluorescent nanospheres.** Top panel: PSF without remote focus arm. Bottom row: PSF obtained with remote focusing arm in place, measured at different z-position (from -26 to 26 microns relative to the nominal focal plane). A conventional, non-resonant galvo was used in the remote focusing arm for these measurements. The table below gives axial and lateral Full Width Half Maximum measurements for each PSF shown. Numbers -5 to 5 refer to the different PSFs shown in the bottom row (mean, n=5). No-RF: PSF obtained without Remote Focus arm in place, i.e. the PSF shown in the top panel.


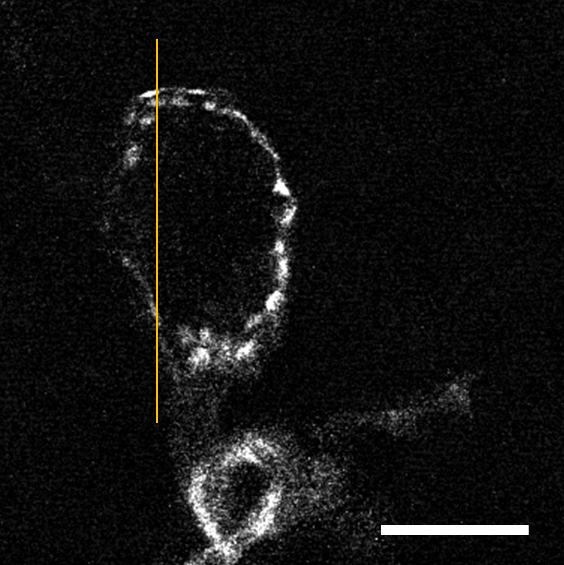


**Supplementary Figure S14** Lateral XY view of the zebrafish heart. Yellow line depicts where the axial XZ view was acquired which is shown in Figure 5h. Scalebar 50 microns.


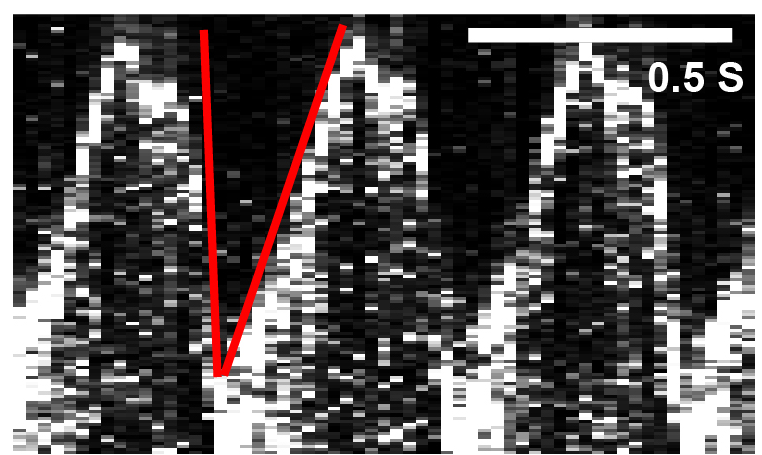


**Supplementary Figure S15** Zoom in of the kymograph shown in Figure 5i. Red lines are visual aids to show the different slopes for the rapid contractions and slower retractions of the heart.


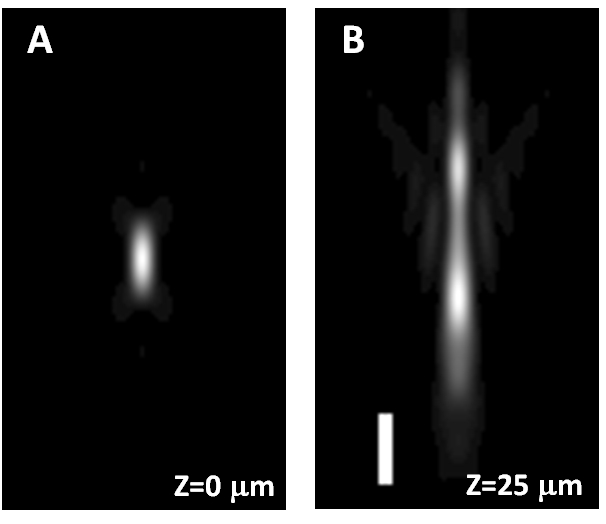


**Supplementary Figure S16** Numerical simulations of the point spread function at nominal focus and defocused by 25 microns using only a quadratic phase function, corresponding to defocusing with an ETL or TAG lens. The axial Full Width at Half Maximum of the PSF increases from 1.1 microns to 4.8 microns. Scale bar 2 microns. The simulation was done using previously published code^2^ for a vectorial model (Debye theory) and assumed a refractive index of 1.33, a wavelength of 900 nm, 2-photon absorption (squaring of the intensity PSF) and a numerical aperture of 1.05.


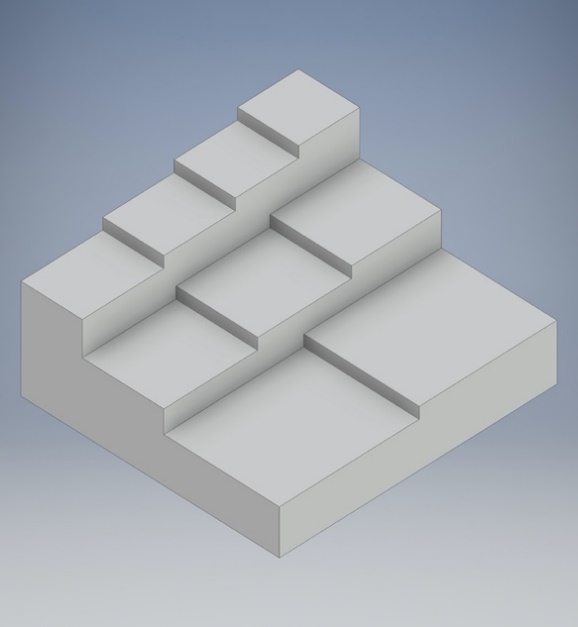


**Supplementary Figure S17** 3D rendering of a two-dimensional stair case mirror with 9 steps. Steps widen with increasing axial distance from nominal focal plane. A two dimensional raster scan over this mirror could address 9 different focal planes.


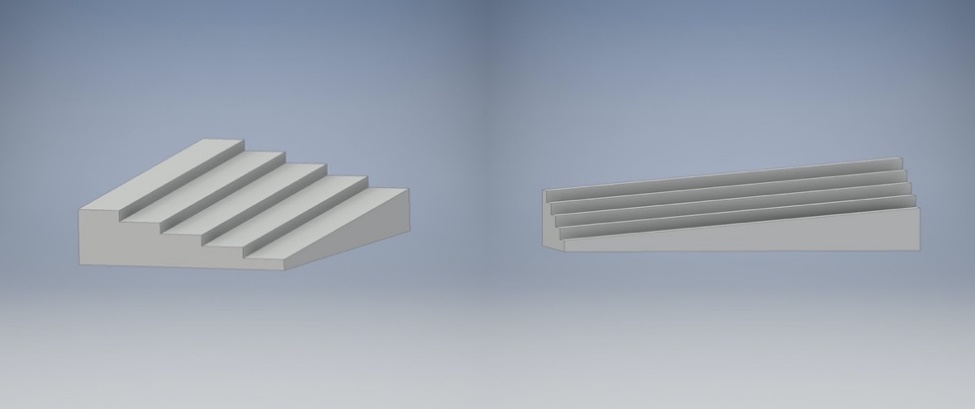


**Supplementary Figure S18** Rendering of a mirror with discrete steps in one dimension and tilted ramps in the other dimension. Scanning along the tilted ramps provides continuous scanning over a limited axial range. By moving the scan to adjacent ramps, a larger total axial range can be accessed with in principle arbitrarily small step sizes.

**Supplementary video**


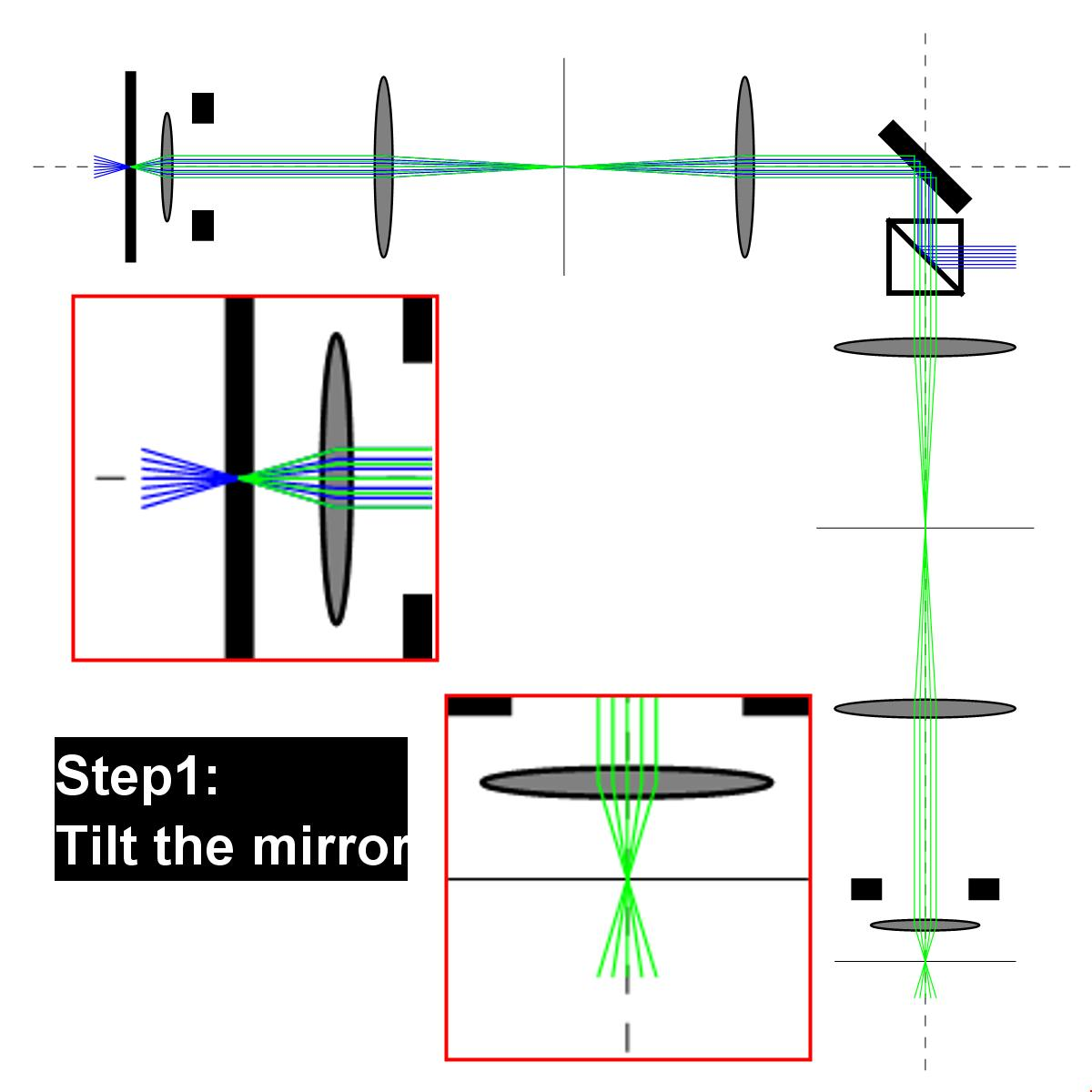


**Supplementary Video 1** Schematic video explaining the tilted mirror remote focusing technique.


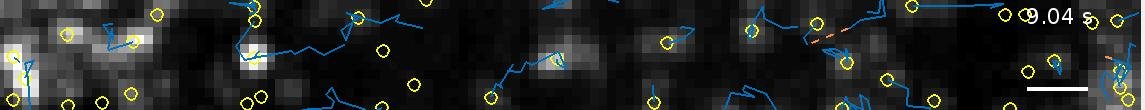


**Supplementary Video 2** Tracking of Genetically encoded multimeric nanoparticles (GEM) inside an MV3 cell, as imaged by ASLM at 20 ms image integration time, and 3.57 volumes per second. Axial YZ view of the perinuclear region. Yellow circles indicate detected vesicles and blue lines illustrate cumulative tracks. Scale bar: 1 microns.


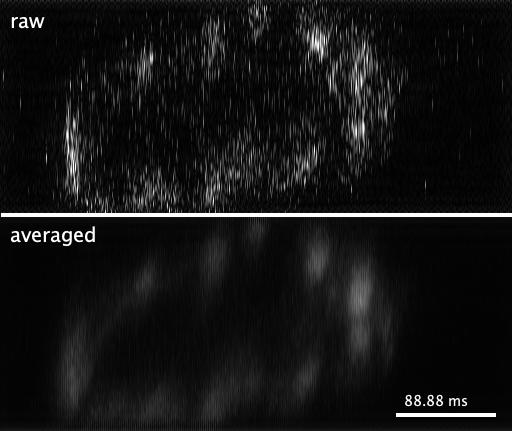


**Supplementary Video 3 Axial imaging of the Zebrafish heart at 45 frames per second.** XZ image as acquired with 2-photon microscopy using resonant remote focusing with a remote mirror tilted by 5 degrees. Top shows raw data, bottom shows data averaged over 30 cycles. Scale bar: 25 microns.


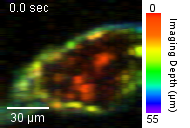


**Supplementary Video 4 Volumetric imaging of the Zebrafish heart at 7.4 volumes per second.** XY image with depth in z color coded, as acquired with 2-photon microscopy using resonant remote focusing with a remote mirror tilted by 5 degrees.


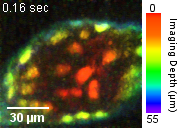


**Supplementary Video 5 Volumetric imaging of the Zebrafish heart at 156 volumes per second.** XY image with depth color coded, as acquired with 2-photon microscopy using resonant remote focusing with a remote mirror tilted by 5 degrees. This movie was reconstructed from 201 consecutively acquired axial XZ timeseries that were acquired over 1s intervals with a 2D frame rate of 156Hz.

**Supplementary References**

1. McGorty, R., Xie, D. & Huang, B. High-NA open-top selective-plane illumination microscopy for biological imaging. *Optics Express* **25** (2017).

2. Manton, J.D. & Rees, E.J. triSPIM: light sheet microscopy with isotropic super-resolution. *Optics Letters* **41** (2016).
